# Prevention and Treatment of Proximate Decay

**Published:** 1875-04

**Authors:** L. G. Noell


### PREVENTION AND TREATMENT OF PROXIMATE DECAY.

BY L. G. NOELL, D. D. S.

*Read before the Mississippi Valley Dental Society, March 4th*, 1875.

A beautiful story is told of how a heroic little Hollander, eight years old, spent a frosty night, with his chubby little hand thrust into a chink of one of the levees that serve to shut off the sea from the shores of Holland; and of how his suffering devotion was promptly rewarded by a grateful people, whose lands he had saved from the dreaded crevasse.

The ready good sense of this heroic child should teach us, as dentists, a useful lesson. Well did he know that the pretty trickling rill which he found would, in a few short hours, grow into a floodgate, through which the resistless sea would pour upon the homes of the sleeping peasants.

A favorite aphorism with a distinguished medical teacher was, "Check the beginnings," and often did he impress upon his students the importance of timely medication in every disease. But this truth, important and beautiful as it is, does not embody the high and holy ambition that should actuate the true physician; his aim should be to *prevent the occurrence of beginnings.* It should be his to guard the citadel of health from every attack of disease. If his aim be lower than this he is undeserving of the high calling of doctor, for the word comes from *doctus*, a teacher. The physician or dentist is not a doctor, unless he be a teacher of hygiene—unless he instruct his patients in the laws of health, and teach them to ward off disease.

As dentists, we need this doctrine forcibly impressed upon us, and if its philanthropic principles could only be carried out to the fullest extent by all, etiological light would break in upon us, and our profession would make grand strides toward the goal of perfection. But it is not our purpose to dwell longer upon truths that have been often set forth by abler pens. We propose to offer a few suggestions as to the manner in which decay is to be prevented and arrested upon the proximate surfaces of the teeth.

There is much that is radical, and we think much that is highly injurious in the system professed by Dr. Arthur, in his work on the above subject; and yet there is in it that soul of truth which is said by philosophers to exist in all error, and which checked and guided by the many criticisms that have been made upon the book will be productive of much good.

It is not our design to pick flaws in Dr. Arthur's work—to notice little contradictions, or complain of its style—we leave this to the critics; but there are one or two points in his teaching against which we do wish to enter our most solemn protest. The first of these is the idea that we are to anticipate decay, and separate teeth whose proximate surfaces are yet smooth, and showing no signs of disease. The enamel does resist decay longer than dentine, notwithstanding his effort to beat around this truth, and was evidently designed by the great Architect to protect the teeth from decay, as well as to furnish a hard triturating surface. Had not this been true, had it only been designed to furnish a hard grinding surface, the arrangement in the case of man—that animal whose teeth are most prone to decay—might have been similar to that in the teeth of the ruminentia. If it is true that the enamel resists decaying agents longer than dentine, it should never, under any circumstances, be disturbed until decay has penetrated it.

The second point to which we would object, is the teaching that separations should be repeated when decay recurs. We think one separation is all that ought ever to be made, nor should that be carried to the extent of disfigurement; it should only be sufficient to secure a permanent opening between the teeth, and when this amount of cutting fails to remove the decay the operator should desist, and proceed to fill. Should the repeated cuttings recommended by Dr. Arthur be made, not only would great disfigurement result, but the teeth would be so crippled as masticators as to be of little value to the patient.

We propose the following course as our conception of that most advisable to be pursued in all cases where full control can be had of the patient from the age of two years up to the period of adolescence. We do not object to dental advice and attention, commencing with the eruption of the central incisors, but it is seldom that parents can be made to appreciate the necessity of this. Indeed, the opinion is generally prevalent that there is no need of a dentist until the shedding of the temporary set has commenced. We would, however, if possible, begin our treatment as soon as the temporary arch is completed, and directing our attention at first to the mother rather than the child, we would instruct her, as well as we could, in the nature and causes of decay, and the prophylactic measures she should adopt to preserve the teeth of her child. We would enjoin upon her the necessity of daily brushing, with a soft brush, and friction upon the gums with a napkin. These implements we would place in the hands of the mother or nurse, with definite instructions how to use them. We would impress upon the mother the necessity of frequent examinations, and explain to her, as clearly as possible, our plan for future proceedings. Let us follow up our hypothetical case, nntil it has reached the period of life when general osseous hardening commences, and dental decay is on the decline.

We fix no limit of frequency of examinations, for some cases will require more and others less attention. Let them be made as often as the exigencies of the case may demand, and at each examination let a thorough and systematic inspection of the teeth be made, upon all of their surfaces. Wherever softening and disintegration of the enamel upon the proximate surfaces has occurred, let them be separated and thoroughly polished. As to the manner in which separations should be made, we have only to say that the spaces should be wide enough to secure a permanent opening down to the gum, and that they should be somewhat wider on the lingual than the buccal side. If bold cavities are revealed, in separating we would prefer to excavate and fill, rather than cut away so much tooth substance as would be sacrificed in an effort to remove every trace of decay. We believe that separations can be made between the molar teeth with less pain and inconvenience, with the corundum disks designed for the engine than with either chisel or file, but these should always be used with the shields for protecting the soft parts. An unfortunate slip in the mouth of one of these timorous little patients may make him ever after unmanageable. With the disk, there need be none of that chafing of the lips that inevitably accompanies the use of the file. The chisel will be found more applicable to the front teeth, in separating which it should always be employed. As to the manner in which they should be cut we shall speak more definitely further on. Cavities upon the crowns of the teeth must, of course, be filled.

As to the materials to be employed in filling children's teeth, it is not the province of this paper to discuss, but we will, in passing, merely indicate our preference for gutta percha. Where, from the behavior of the patient, a more durable filling may be desired, we would choose gold, believing it as easy of manipulation as tin. We would particularly recommend it for filling crown cavities in the deciduous teeth, for gutta percha does not here well withstand the friction of mastication. We believe the gutta percha will be found sufficiently durable, however, in all simple proximate cavities of the deciduous teeth—in all cases where the enamel is not broken down from the crowns, rendering the cavities compound. We will seldom have this complication to contend with, where we have our patients fully under control, and where frequent examinations are made.

Thus we would watch over the deciduous teeth during their development, and endeavor by preventive as well as operative measures to retain them, until their allotted work should be done, and they should be eliminated by nature's original method—*i. e.*, by the absorption of their roots. We come next to the treatment of the permanent teeth, and now much of the difficulty which surrounded our work in the beginning has been removed, and we see our way more clearly to the end in view.

Our little patient can now be appealed to as an intelligent being, and while we do not at first dispense with her aid, we do not rely solely upon the mother for the execution of our orders.

The first teeth in the permanent set demanding our attention are the sixth year molars. In reference to these teeth we can not do better than to fully indorse the treatment which Dr. Arthur recommends. His plan is to cut away a portion of the distal surface of the second temporary molar as soon as the crown of the permanent tooth is fully formed. We do not object to this anticipation of decay in this instance, because the temporary tooth has now served out half of its term, and we would be willing to sacrifice a part of its grinding surface, in order to preserve the integrity of the enamel upon the sixth year molar. In making this separation, however, we must employ the chisel or safe-sided file, instead of the disk, for it is important to avoid injuring the enamel upon the permanent molar, as long as it is free from caries. The practice of extracting the sixth year molars at an early age, with a view to gaining space between the remaining teeth, thereby lessening their liability to decay, has been strongly recommended. Their extreme liability to decay, the crowded condition of the wisdom teeth, when they are retained, and the space gained for all occupants of the dental arch by their extraction, are points that have been urged with much speciousness by the advocates of this practice. We would never advise their removal unless their pulps should become exposed by caries, prior to the age of fourteen, in which case no attempt should be made to fill them, provided the bicuspids are free from decay, or in a salvable condition. When the pulps of the first permanent molars are found dead, or when the chances for preserving their vitality are meager, we would advise extraction, even as late as the age of seventeen. If from the nature of the case we should deem it advisable to extract any of the teeth to secure room, we would prefer to remove bicuspids, believing the first molars to be more useful masticators, and less liable to be attacked by caries than the bicuspids after the age of twenty-one.

The question may be asked, Would you, in case the teeth be regularly arranged and free from caries, recommend the extraction of a pair of bicuspids, above and below, for the purpose of gaining room and preventing decay?

We answer, very emphatically, no. Though in doing so we must differ with a gentleman for whose opinion we have a high regard.

If from marital misadaptation the patient should inherit large teeth with small jaws, and marked irregularity should exist, we would strongly recommend extraction, and our selection would be from the bicuspids, allowing the condition of the case to determine whether we should remove the first or second.

The next spaces demanding our attention, will be those between the superior incisors. The inferior incisors are so generally exempt from decay that ordinary attention to cleanliness will, in the majority of cases, suffice for them.

We may now address our instructions to both mother and child, and in addition to the daily brushing and friction with the napkin, previously enjoined, we would advise the daily passage of floss silk through the spaces between all of the teeth. If these instructions are properly given and faithfully observed. in few instances will it be found necessary to separate or fill the incisor teeth upon their proximate surface. Of course there will be cases where the constitution is so feeble, and the oral fluids so vitiated by disease that no amount of hygienic care will preserve the teeth from decay; but even in the frailest organizations much can be done to stay its progress by persistence in preventive measures.

If it is found necessary to separate between the incisor teeth—and this should not be resorted to unless the enamel is already softened by decay—the chisel will be found a valuable instrument to perform the heaviest part of the work, and we prefer to follow it with an oval, tapering file, finely cut. The spaces should be left wider at the lingual than the labial opening; indeed, the labial plate of enamel should be left untouched, if possible. The chisel should be permitted to do its deepest cutting at the point of deepest softening, coming out above the gum.* This will leave the opening in the best shape for finishing with the oval file and polishing tape. Under no circumstances should the chisel be carried through to the neck of the tooth, but a bulging portion of the crown is to be left, to prevent the approximation of the teeth. It will generally be found best to wedge apart, before commencing the operation. As we have already remarked, the space should only be sufficiently wide to remove the softened structure, and preserve a permanent opening. If deep cavities are revealed, rather than sacrifice the symmetry of the teeth in an effort at removal, we would desist and fill. Of course, all we have previously said of the importance of preserving the symmetry of the teeth applies with especial force to the permanent incisors. Owing to the double V shaped space, existing in the enamel contact of the next teeth, that will claim our attention—the bicuspids—it will be found more difficult to prevent them from being attacked on their proximate surfaces. As soon as softening is detected, they are to be separated, leaving the space widest at its lingual opening.

The buccal and lingual angles are to be rounded off, the surface thoroughly polished, and attention to cleanliness enjoined.

*As we are speaking of the superior incisors, it would perhaps be better to say below the gum.

Probably the next spaces requiring attention will be those between the second bicuspids and first molars. In course of time separation may be required between the first and second molars, canines and lateral incisors, and ultimately it may be between the second and third molars. In making all these separations we would observe the principles already given.

Of course, all crown cavities should be filled as soon as detected.

